# Oral HPV-associated dysplasia: is koilocytic dysplasia a separate entity?

**DOI:** 10.3389/froh.2024.1363556

**Published:** 2024-02-16

**Authors:** Gabriela Anaya-Saavedra, Marcela Vázquez-Garduño

**Affiliations:** Oral Pathology and Medicine Postgraduate Program, Health Care Department, Metropolitan Autonomous University, Mexico City, Mexico

**Keywords:** HPV, oral dysplasia, koilocytic dysplasia, oral cancer, p16

## Abstract

Oral epithelial dysplasia associated with high-risk HPV infection has received different names since its initial description, such as oral Bowenoid lesions, HPV-associated intraepithelial neoplasia, and oral koilocytic dysplasia. Some features, identified in more or less quantity in some of the descriptions, like apoptotic keratinocytes, karyorrhexis, and mitosoid figures, are intricately connected to viral transcriptional status and, consequently, viral load. Since the variety in terminology has introduced diagnostic confusion within medical and research communities, establishing a uniform and standardized approach to diagnosing HPV-oral epithelial dysplasia is crucial for accurate and early diagnoses and holds significant implications for patient outcomes, particularly in high-risk individuals.

## Introduction

The Greek term dysplasia, denoting abnormal tissue growth, was initially introduced in the context of cervical intraepithelial neoplasia, and is distinguished by the distortion of cellular uniformity and architectural structure in a particular tissue (1). In the realm of cytological changes, particularly in smears, the appropriate term to employ is “atypia”, as opposed to “dysplasia” (2).

The progression of the oral carcinogenic process unfolds through a series of stages, beginning with epithelial hyperplasia. This progression can traverse various grades of epithelial dysplasia, ranging from mild to severe or, as currently designated, from low- to high-grade squamous intraepithelial lesions (3, 4). Oral epithelial dysplasia (OED) stands out as a well-established risk factor for developing of oral cancer, exhibiting a 12% malignant transformation rate, with a range from 0% to 36%, depending on the severity of dysplasia (5–7).

The diagnosis of OED is inherently subjective, related to the intra- and inter-observer variability (3, 8). Over the years, numerous efforts have been undertaken to pursue our philosopher's stone—a biomarker capable of identifying cases fated for progression into oral squamous cell carcinoma (OSCC), to facilitate timely intervention and mitigate comorbidities associated with oral malignancy. Among the biomarkers under investigation, human papillomavirus (HPV) infection has been a focal point of research over the past four decades.

### HPV and carcinogenesis

The role of HPV in oral carcinogenesis has generated considerable debate within the scientific community. Nowadays, it is recognized that merely detecting HPV-DNA in an oral sample is not sufficient to establish HPV as a carcinogenic agent, as the presence of HPV DNA may signify a transient infection. The potential significance lies in the presence of episomal HPV-DNA, which has been implicated in the malignant transformation of oral epithelial dysplasia (9).

In contrast to the unequivocal causal role of HPV in anogenital and oropharyngeal carcinogenesis, the prevalence of high-risk HPV (HR-HPV) in OSCC has been reported in a wide range from 0% to 58% (10, 11). Adding complexity to this variability is the ongoing controversy surrounding the prognosis of patients with HPV-related oral cancer. While specific studies find no discernible differences (12, 13), others assert significantly worse survival outcomes for patients with HPV-16 non-associated OSCC compared to their HPV16-positive counterparts (14).

Moreover, there is a persistent debate about the role of p16, a tumor suppressor protein, as a surrogate marker for HPV infection. While p16 is a valuable tool for identifying HPV infection in anogenital and oropharynx areas, its utility in studies focused on OED and OSCC is debated. Some studies report high sensitivity (15, 16), while others assert its null effectiveness in HPV identification (13, 17). The diverse definitions of p16-positivity further contribute to potential misinterpretations of this biomarker's utility (10).

### HPV-oral epithelial dysplasia

In this context, a distinct subset of OED, linked to HR-HPV (mainly HPV-16), has been identified and characterized by specific histological features (16, 18, 19). While epidemiological data on its prevalence is lacking, McCord and Bradley (16) suggest that approximately 18% of severe oral epithelial dysplasia cases are associated with biologically significant HR-HPV infection. Table 1 provides a comprehensive overview of studies focused on HPV-oral epithelial dysplasia, the clinical and histological characteristics, and the HPV prevalence informed.

**Table 1:** Characteristics of the studies regarding the prevalence of HPV-DNA and p16 immunoexpression in histologically confirmed oral epithelial dysplasia.

	Type of study	*n*	Males	Mean age (years)	Anatomical sites	Clinical appearance	Histologic features	Histological diagnosis	HPV	IHQ p16+
Fornatora et al. (20)	Retrospective (1986–1995)	48*4 PVV	84%	39 (21–65)	Tongue, lips, and oral mucosa	Fiat or slightly elevated white lesions	Variable findings according to dysplasia grade. Acanthosis, orto/parakeratosis, anastomotic rete pegs, hyperchromatic nuclei and mitosoid figures. Koilocytosis and binucleated cells.	KD (64%) COED (36%)	ISH KD (81%) COED (0%)	—
Daley et al. (21)	Case series	7	100%	47 (29–69)	Tongue (28%) Buccal mucosa (28%)	Leukoplakia (71%)	Large, sometimes multinucleated, atypical and dyskeratotic cells or apoptotic fragments, atypical mitoses scattered throughout the epithelium.	HG-D (57%)	ISH (71%)	—
McCord et al. (16)	Retrospective (2007–2009)	40HG-D	57%	59 (15–84)	Tongue 40% Floor of the mouth 40%	NS	Diffuse, full-thickness loss of squamous differentiation. Mitotic figures, multinucleated cells, and dyskeratosis. Absence of koilocytosis, no fit KD criteria.	HG-D (52%) LG-D (48%)	ISH (17.5%)	27%
Woo et al. (19)	Bidirectional (2008–2012)	20	85%	56 (41–71)	Tongue, buccal mucosa.	Leukoplakia (85%)	Epithelial hyperplasia with marked karyorrhexis and apoptosis. Scattered koilocytes.	HPV-associated oral intraepithelial neoplasia.	ISH (100%)	100%
Chen et al. (22)	Cross sectional	59	50%	55 (28–79)	Tongue (25%) Buccal mucosa (30%)	Leukoplakia (74%)	NS	NS	RT-PCR + DS (0%)	—
Khanal et al. (23)	Retrolective (2003–2015)	38	82%	>55: 52%	Lateral tongue or floor of the mouth (52%)	White or red/white plaque variably rough/papillary surface	Dysplasia signs plus variable amounts of karyorrhectic and apoptotic cells.	HG-D	PCR + DS (8%)	36%
Zhang et al. (24)	Retrolective (2012–2015)	10	70%	63 (45–71)	Floor of the mouth (60%)(include oropharyngeal sites)	NS	Dysplastic cells with oval to spindled nuclei, high nuclear to cytoplasmic ratios,indistinct cell borders and little to no surface maturation.	HG-D/CIS	ISH (100%)	100%
Lerman et al. (18)	Case series (2008–2016)	53	89%	55 (41–81)	Tongue and floor of the mouth	Leukoplakia (62%)	Parakeratosis (94%), karyorrhexis and apoptosis throughout the hyperplastic stratified squamous epithelium.	HG-D (85%) OSCC (15%)	ISH (91%)	100%
Saleh et al. (25)	Case report	1	Male	57	Multiple lesions in buccal mucosa, tongue and palate	Leukoplakia	Morphologic and cytologic severe dysplastic features plus mitosoid bodies.	Severe dysplasia	—	Positive
Alsabbagh et al. (26)	Retrospective (2013–2017)	12	100%	57 (50–94)	Floor of the mouth (25%) Tongue (25%)	Leukoplakia	Karyorrhexis, apoptotic cells, ortho/parakeratinization, The remainder of the cells were basaloid, showing “wind-blown” atypia.	Mild to moderate (8%) Severe/CIS (92%)	RT-PCR (100%)	100%
Erira et al. (27)	Cross sectional	30	21%	NS	Tongue (43%)	Leukoplakia (37%)	Variable findings according to dysplasia grade.	Mild (43%) Moderate (47%) Severe (20%)	PCR (43.3%)	–
Hendawi et al. (28)	Cohort (2014–2016)	33	60%	63 (36–87)	NS	Leukoplakia in most cases	Characteristics of OED, apoptotic bodies, abnormal mitotic figures which can be difficult to distinguish.	Severe epithelial dysplasia	ISH HR-HPV: (18%)	HPV+:100% HPV-: 18%
Tomo et al. (17)	Cross sectional (2011–2017)	50	62%	59	NS	NS	Variable findings according to dysplasia grade.	No dysplasia (68%) Mild (16%) Moderate (12%) Severe (4%)	Linear array (0%)	Low: 60% High: 40%
Argyris et al. (29)	Retrospective	13	92%	62.8 (51–87)	Floor of the mouth (31%) Tongue (31%)	NS	Variable findings according to dysplasia grade.	Oral epithelial dysplasia.	IHC HPVE7 (100%)	100%
Sri et al. (30)	Cross sectional comparative (2010–2012)	20	NS	NS	NS	Leukoplakia (50%)	NS	Mild (30%) Moderate (50%) Severe (20%)	PCR (5%)	–
Jawahar et al. (31)	Retrospective	30	97%	>60: 63%		Homogeneous leukoplakia (93%)	Variable findings according to dysplasia grade.	Mild (30%) Moderate (57%) Severe (13%)	IHC-E6 (36%)	10%
Roza et al. (32)	Case series (2011–2022)	5*4 PVV	80%	55 (51–60)	Buccal mucosa (60%)	Leukoplakia (60%)	Karyorrhectic cells (mitosoid bodies) and apoptotic keratinocytes with dense eosinophilic cytoplasm.	Severe dysplasia	ISH (80%)	100%

HPV, human papillomavirus; KD, koilocytic dysplasia; COED, conventional oral epithelial dysplasia; HG-D, high grade dysplasia; LG-D, low grade dysplasia; CIS, Carcinoma *in situ.* OSCC, Oral squamous cell carcinoma; PCR, polymerase chain reaction; DS, Direct sequencing; RT-PCR, reverse transcriptase polymerase chain reaction; ISH, *In situ* hybridization; IHC, Immunohistochemistry; PVV, People living with HIV; NS, not specified.

In their report from 1986, Fornatora et al. (20) introduced a distinctive variant of oral epithelial dysplasia that, based on light microscopic features, appeared to harbor HPV. These lesions demonstrated concurrent histologic features of cytopathic damage, including acanthosis, koilocytosis, and keratinocyte multinucleation, in addition to conventional OED characteristics such as basilar hyperplasia and nuclear pleomorphism. Previously, a crucial manuscript by Koss and Durfee (2), preceding the molecular biology and virology era, set the basis for unveiling HPV infection's role in the etiology of cervical cancer. They described large cells with irregular, hyperchromatic nuclei surrounded by clear cytoplasm, coining the term “koilocytotic atypia” from the Greek “*koilos,*” meaning hollow or cavity. Intriguingly, these cells were initially identified in uterine cervical smears before tissue recognition, preceding their identification in the oral mucosa. Building on the pioneering work of Koss and Durfee (2), Fornatora et al. (20) brought the term koilocytic dysplasia to the oral mucosa, outlining a distinct subtype of oral epithelial dysplasia characterized by unique clinical and histologic features indicative of the presence of HPV-DNA.

Later, Daley et al. (21) revising previous studies, reported seven cases of oral lesions exhibiting bowenoid histological alike features to Bowen's disease, a solitary, irregular, erythematous macule of the skin or glans penis (erythroplasia of Queyrat), that histologically exhibits carcinoma *in situ* (CIS), characterized by disordered maturation and scattered large and atypical cells, and mitosis throughout all layers of the epithelium. These oral lesions displayed various histologic features and biological behavior, suggesting an association with the p53/WAF-1 apoptotic pathway.

In subsequent years, studies analyzing HPV prevalence in OED were published, leading to two meta-analyses in 2011 (33, 34). Jayaprakash et al. (33) reported an overall prevalence of HPV-16/18 in OED of 24.5% (CI: 16.4–36.7), with a threefold increase in OED compared to normal biopsies. However, no differences in HPV-16/18 between dysplastic lesions and cancers or between mild, moderate, or severe dysplastic lesions were found, supporting the assumption that HR-HPV infection occurs during the early phase of oral carcinogenesis. Likewise, Sirjänen et al. (34) demonstrated a significantly increased risk of HPV among individuals with OED when compared to controls, presenting a pooled estimate across all studies of 3.87 (95% CI 2.9–5.2).

In 2013, McCord et al. (16) revisited the term koilocytic dysplasia. They conducted a retrospective study involving immunohistochemical (IHC) staining for the p16 protein and *in situ* hybridization (ISH) for HPV-DNA in 40 high-grade and 37 low-grade dysplastic samples. Within this cohort, they identified a small subset of OEDs (*n* = 7) associated with HR-HPV infection. These cases exhibited distinctive features, including a loss of squamous differentiation, abnormal proliferation indicative of the oncogenic effects of high-risk HPV, mitotic-like structures, multinucleated cells, and dyskeratotic cells throughout the epithelial thickness—reminiscent of Bowen disease of the skin. Integrating micromorphological findings with molecular results, they underscored a clear correlation between HPV detection and histomorphology. Notably, they acknowledged their cases did not align with the criteria for koilocytic dysplasia described in 1956 by Koss and Durfee (2) and reiterated by Daley et al. (21) under the term “oral Bowenoid lesions.”

In the same year, Woo et al. (19) presented the findings of a study involving 20 cases of epithelial dysplasia characterized by a substantial presence of apoptotic cells. Their observations included karyorrhexis and apoptosis, featuring brightly eosinophilic apoptotic cells distributed throughout the epithelial thickness. The apoptotic cells were surrounded by keratinocytes displaying conventional dysplastic changes. Although *in situ* hybridization studies confirmed the presence of HR-HPV in all cases, the authors noted the scarcity of typical koilocytes when using the rigorous criteria of peri-nuclear halos and nuclear enlargement. Consequently, they proposed the term “HPV-associated intraepithelial neoplasia” to maintain nomenclature consistency with HPV-associated lesions in the lower anogenital tract.

Subsequently, the study by Zhang et al. (24) broadened the understanding of HPV-associated oral epithelial dysplasia by introducing a novel nonkeratinizing pattern of severe dysplasia/CIS. The analysis involved 98 patients diagnosed with severe dysplasia/CIS, revealing that 3% exhibited a nonkeratinizing histological type. This subtype was characterized by dysplastic cells with oval to spindled nuclei, high nuclear-to-cytoplasmic ratios, indistinct cell borders, and limited surface maturation. Notably, most non-keratinized cases were predominantly located in the oropharynx and subglottic area, where epithelia either include or resemble transitional epithelium.

The significant diversity in histopathological characteristics linked to dysplasia grade has resulted in using multiple terms such as oral koilocytic dysplasia, oral Bowenoid lesions, oral intraepithelial neoplasia, and HPV16-specific dysplasia. The histological presentation of HPV-associated dysplasia is intricately connected to the viral transcriptional status and the number of viral copies; thus, it is reasonable to expect that lesions may or may not exhibit koilocytosis and varying degrees of apoptosis. In addition, using the term “intraepithelial neoplasia” might lead to confusion, especially among surgeons, potentially resulting in more aggressive management than necessary.

**Figure 1:**
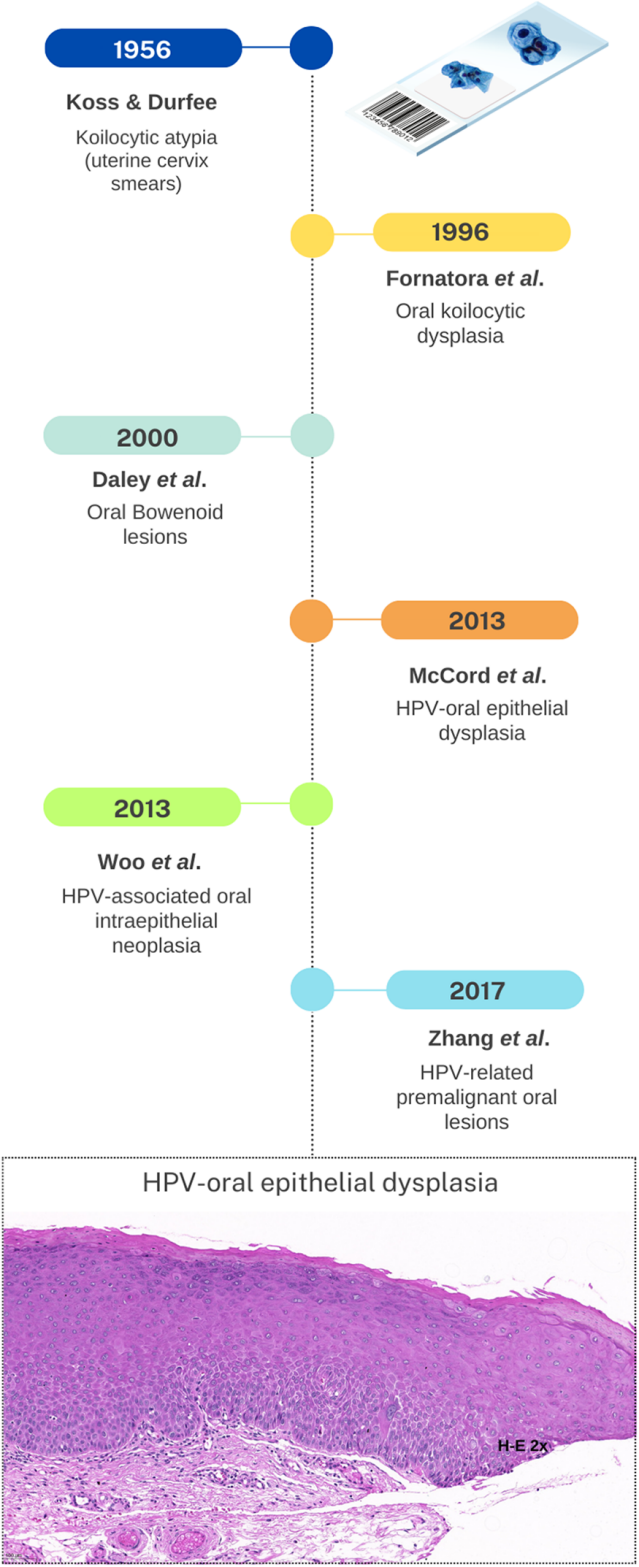
Timeline showing the evolution of the term HPV-oral epithelial dysplasia, from its adoption from cervical cytological samples, to the present. The histopathological image at the end shows one of the many faces of HPV-OD (Archives of the laboratory of Oral Pathology of UAM-X, Mexico).

Therefore, to mitigate potential confusion and ensure appropriate treatment strategies, we propose adopting the unified term “HPV-oral epithelial dysplasia” to this entity. A standardized nomenclature aims to enhance clarity, facilitate accurate communication, and promote a cohesive understanding of this distinct subset of oral epithelial dysplasia associated with high-risk HPV infection (Figure 1).

### Clinical and histopathological findings of HPV-OED

As described in Table 1 and verified by prior systematic reviews and meta-analyses (33–35), HPV-oral epithelial dysplasia exhibits a preference for males, mainly manifesting after the sixth decade of life. The lesions were predominantly consistent with leukoplakia and located in the tongue, buccal mucosa, and the floor of the mouth.

Microscopically, HPV-OED stands out markedly from conventional severe oral dysplasia due to its distinctive full-thickness basaloid morphology. The basal layer displays hypercellularity, condensed coarse chromatin (reflecting degenerate mitoses), occasional multinucleation, dense eosinophilic cytoplasm, a high nucleus-to-cytoplasm ratio, and a blurred boundary between the basal and spinous layers, resulting in the dark basaloid cell morphology. Another essential feature is the pronounced epithelial hyperplasia with deeply invading rete ridges, presenting a corrugated eosinophilic parakeratin or orthokeratin surface. A frequent observation is the presence of sharply defined lateral borders between dysplastic and normal epithelium (8, 32).

Although the presence of karyorrhectic and apoptotic keratinocytes typically located within the superficial layers of the epithelium has been described as a surrogate microscopic feature for HPV-OED (19, 20, 32, 35), some studies contend that koilocytes and multinucleated keratinocytes are inconspicuous and encountered only occasionally (16, 18, 23), recommending further confirmation of HPV infection in routine practice (32).

Interpretation of abnormal nuclear morphology, including karyorrhexis and mitosis figures, is subjective, challenging to distinguish, and occasionally overlaps with those observed in severe epithelial dysplasia (28). These features have demonstrated poor performance as a standalone test, underscoring the insufficiency of relying solely on specific histological features (28). In simpler terms, the presence of karyorrhexis and apoptotic bodies is not universal in all cases of HPV-oral epithelial dysplasia. Conversely, not all conventional cases of OED displaying these characteristics are necessarily associated with viral cytopathic damage.

Hence, recent publications propose refraining from applying grading criteria for cytological and architectural features of conventional OED to HPV-OED, given its unique etiology and morphology (8, 32). In particular, the involvement of the full thickness of the epithelium does not inherently signify severe dysplasia in terms of its risk of malignant transformation (8).

### HPV in OED

While most studies on HPV-OED employed DNA *in situ* hybridization for identifying high-risk HPV (HR-HPV) (16, 19, 21, 24, 28, 29, 32), others employed diverse methods such as polymerase chain reaction (PCR) (27, 30), real-time PCR (RT-PCR) (26), and even DNA sequencing (22, 23). Despite their high sensitivity, it's important to note that a positive test from these assays might signify sample contamination or a low-level transient infection rather than the presence of active high-risk HPV.

Studies on oropharyngeal squamous cell carcinoma have suggested that the most effective stratification for detecting high-risk HPV is through RNA RT-PCR, RNA ISH, and p16 immunohistochemistry (32). Taking this into consideration is advisable for a more accurate assessment of active high-risk HPV presence.

The HPV oncoproteins E6 and E7 can potentially disrupt the activity of tumor suppressor proteins p53 and pRB within the cell cycle, leading to malignant transformation and facilitating uncontrolled proliferation (27, 36), even at early stages (23). Therefore, the expression of HPV-E6 is considered a more valuable diagnostic test, demonstrating higher specificity for detecting high-grade oral epithelial dysplasia than the sole detection of HPV-DNA (31).

Based on a systematic review encompassing 31 studies (832 cases) conducted by de la Cour et al. (35), the overall pooled prevalence of HPV DNA in oral epithelial dysplasia was determined to be 27.2% (95% CI: 17.6–38.1). A sensitivity analysis focusing on 14 studies, which included a control group, revealed an overall pooled HPV-DNA prevalence in oral dysplasia of 32.6% (95% CI: 18.1–49.0). The pooled HPV DNA prevalence among control subjects was 11.1% (95% CI: 3.5–22.2).

Similarly, findings from Jayaprakash et al. (33) indicate that the presence of HR-HPV in one-fourth of HPV-OED aligns with the hypothesis that HPV plays a substantial role in the early phases of oral and oropharyngeal carcinogenesis. However, it is crucial to underscore that detecting HPV alone does not establish a causal association, as it can also be identified in normal oral tissue.

The pursuit of HPV testing is expressly advised in instances where histological evidence strongly indicates HPV-OED. Screening for HPV infection in isolation is discouraged due to an imperfect balance of advantages and potential drawbacks (37). This imbalance is primarily attributed to the transient nature of most oral HPV DNA, increasing the probability of false positives. Hence, it underscores the significance of adopting a prudent and contextually informed approach to HPV testing in assessing oral dysplasia.

### P16 immunoexpression

Immunohistochemistry has been widely used to assess p16 immunoreactivity as a reliable surrogate marker of HPV in anogenital and head and neck cancers (17). Despite its utility, conflicting data has emerged, suggesting a notable risk of false-positive results in the context of HPV-related oral lesions. This discrepancy is attributed to the need for a standardized cut-off for interpreting p16 immunoreactivity in the oral cavity, contributing to challenges in its accurate application.

Moreover, previous studies, including those by McCord and Bradley (16), Khanal et al. (23), and Buajeb et al. (38), have reported that p16 immunoreactivity is infrequent or nearly absent in oral dysplastic lesions. These findings underscore the complexity of relying solely on p16 as a biomarker for HPV-associated oral dysplasia.

While most studies on HPV-OED have reported typical diffuse and strong p16 positivity, recognizing this biomarker as a valuable predictor of the presence of transcriptionally active high-risk HPV infection (18, 19, 24, 26, 28, 29, 32), other investigations have observed lower immunoreactivity in HPV-OED cases (31) or elevated p16 levels in HPV-negative oral epithelial dysplasia samples (17).

The varied outcomes in p16 immunoreactivity across different studies emphasize the need for a standardized approach to defining positivity, prompting further investigation into its specificity and sensitivity in the context of oral dysplastic lesions. In addition, it is crucial to highlight the importance of a cautious and comprehensive evaluation when interpreting p16 immunoreactivity results in the diagnostic and prognostic assessment of HPV-related oral epithelial dysplasia.

### Prognosis of HPV-OED

While the progression characteristics are not fully elucidated, some authors (19, 35) suggest that HPV-associated oral dysplasia has the potential to progress into HPV-associated oral cancer. Nevertheless, conservative surgical excision appears curative in most cases, demonstrating no signs of recurrence after an average follow-up of 39 months (32). Notably, Allam et al. 2008 (39) reported promising results with imiquimod, an immunomodulatory drug successfully used to treat HPV infections in the anogenital area.

Additionally, a potential contributing factor to the progression of HPV-OED to cancer could be the microbiome, a complex ecosystem of microorganisms that has been implicated in the advancement of HPV infection to cancer in other HPV-related carcinogenesis contexts (40). Understanding the interplay between the oral microbiome and HPV-associated dysplasia is crucial for elucidating the underlying mechanisms and identifying potential therapeutic targets. Further research should explore and dissect the intricate relationships between the microbiome and HPV-associated oral dysplasia to provide comprehensive insights into the factors influencing disease progression and potential avenues for intervention.

We consider the need to improve the diagnosis of HPV-OED, particularly among high-risk patients, to facilitate early intervention and offer a critical advantage in managing this condition. HPV-OED has been documented in people living with HIV (20, 32) and in recipients of allogeneic hematopoietic stem cell transplantation (HSCT) (40), thus, regular screening for potentially malignant disorders identification is critical. It underscores the importance of integrating HPV-OED surveillance into the routine care of immunocompromised individuals.

Moreover, a key consideration is the role of HPV vaccination in mitigating the currently low incidence of HPV-OED and reducing the associated risk of cancer in these patient populations (8, 25), contributing to a comprehensive strategy for minimizing the impact of HPV-OED, ultimately advancing the well-being of at-risk individuals.

## Conclusions

• Clinically, it is not possible to distinguish between conventional OED and HPV-OED.
• HPV-OED is typically distinguishable from conventional OED on histopathologic grounds, representing a minority of cases.
• A minority of severe dysplasia cases was identified to contain transcriptionally active HR-HPV, emphasizing the need for targeted investigation in this subset.
• While histological characteristics are considered hallmarks, they are not deemed essential for predicting HPV status in OED, underscoring the complexity of the disease.
• The presence of karyorrhexis and apoptotic bodies is associated with HPV status in OED. Yet, their use as a predictive marker needs to be more robust, necessitating further exploration of more reliable indicators.
• p16, often utilized as a biomarker, is found to be insufficiently robust for predicting HPV status in OED, highlighting the need for alternative and more accurate molecular markers.
• Clinical monitoring and extensive molecular studies within this subgroup are imperative to unravel how HPV initiates or influences the progression of OED to oral cancer.
• The multifaceted nature of HPV-OED demands a nuanced understanding of its molecular underpinnings and clinical implications, guiding the development of more precise diagnostic tools and targeted interventions for this challenging condition.
